# Retained Intra-Abdominal Surgical Clamp Complicating Emergency Laparotomy: Incidental Finding on Hysterosalpingogram for Evaluation of Tubal Infertility

**DOI:** 10.1155/2014/963454

**Published:** 2014-11-09

**Authors:** Adebiyi Gbadebo Adesiyun, Nkeiruka Ameh, Hajaratu Umar-Sullayman, Solomon Avidime, Rabia't Aliyu

**Affiliations:** Department of Obstetrics & Gynaecology, Ahmadu Bello University Teaching Hospital, P.O. Box 204, Zaria, Kaduna, Nigeria

## Abstract

The finding of intraperitoneal foreign body complicating surgical intervention broadly remains as an issue of safety in the operative room, a source of emotive concern for the patient, and an upsetting but equally embarrassing situation to the surgeon and the team. However, in the media world, it is a source of sumptuous and captivating headline on the newspaper and to the legal profession, an attractive case to prosecute. A middle age teacher presented with secondary infertility. She had emergency laparotomy fifteen years ago for ruptured tubal ectopic pregnancy in a private hospital and postoperative period was uneventful. Amongst other investigations to find out the cause of infertility, she had hysterosalpingography and a radio-opaque clamp was visualized on the films. She was counselled and had laparotomy. A pair of surgical Kocher clamps was retrieved buried in the mesentery.

## 1. Introduction

Infertility is a common gynaecological condition in sub-Saharan Africa and tubal occlusion from infection related causes is a leading cause of infertility. Hysterosalpingography is the most common preliminary method of assessing the patency of the fallopian tube in most resource constrained medical facilities. Forgotten foreign body at surgery, better referred to as retained surgical item (RSI), may not be uncommon [1], although, when detected in the developing world, it is usually shrouded in secrecy because of fear of medicolegal prosecution and punishment. However, in the developed world, like in the USA and UK, it is a reportable and documentable occurrence [2, 3]. As a result of the aforementioned, the incidence of RSI is hardly reported and fraught with inaccuracies if there was a quote that emanated from the developing countries; this is mainly due to issues with medical data sharing especially when it has to do with complication or negligence. On the contrary, an estimate of 1500 to 2000 cases of RSI occurs on a yearly basis in the USA [4]. Incidence may be lower in laparoscopic compared to laparotomic surgery (open access surgery) and in elective surgery compared to emergency surgery [5–7]. Minimally invasive surgery (MIS) may not be an absolute solution to occurrence of RSI, as Gibbs reported four cases of retained surgical sponge complicating MIS [4].

## 2. Case Report

**Figure 1:**
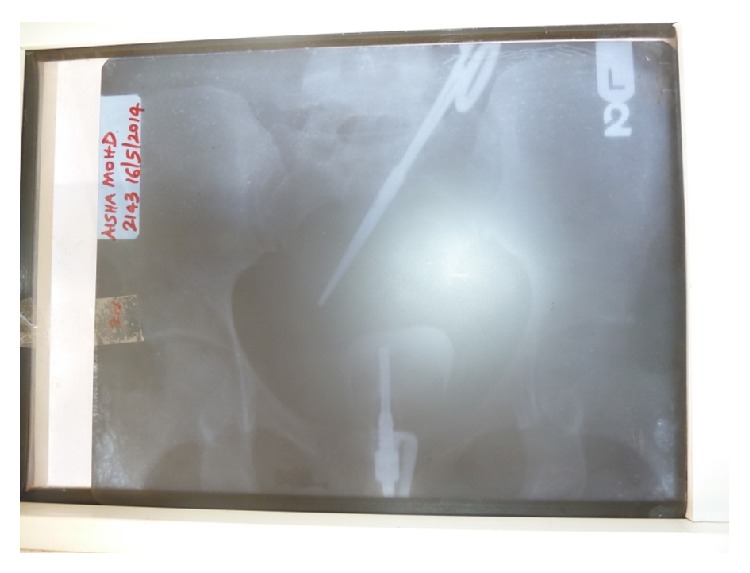
Hysterosalpingogram showing retained surgical clamp.

**Figure 2:**
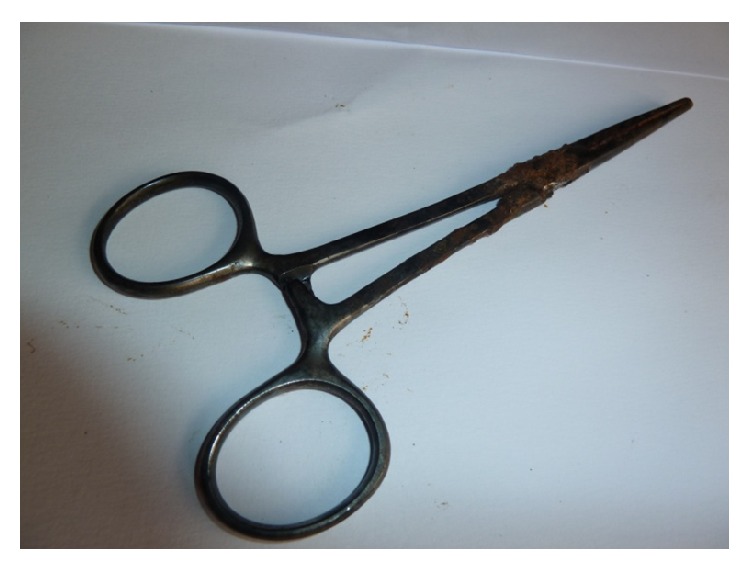
Retrieved Kocher's surgical clamp.

A 42-year-old para 1 + 1 teacher presented with 11-year history of infertility. Her last child birth was in 1999; pregnancy was normal and she had a spontaneous vaginal delivery which was uneventful. However, she lost the child at age of two years. In 2000 she underwent an emergency laparotomy for ruptured tubal left ectopic pregnancy in a private hospital. The postoperative period was uneventful and she was discharged on the fifth day following surgery. She did have a painless three-day normal flow menstruation monthly. Clinical examination did not reveal any abnormality except for obesity (body mass index of 36.2 kg/m^2^) and a midline subumbilical scar from previous laparotomy. Investigations to evaluate the cause(s) of infertility were ordered. On the hysterosalpingogram, features of a metallic clamp in the lower abdominal cavity with the clamp handle at the level of the left transverse process of the fourth lumbar vertebra (Figure 1). Other findings were an irregularly outlined small sized uterine cavity and absence of both tubal outlines. Patient was counselled and worked up for elective laparotomy. Intraoperative findings are pelvic adhesions, fixed uterus, and Kocher clamp entangled within the mesentery. The foreign body was carefully dissected from surrounding tissue and retrieved (Figure 2). She had an uneventful postoperative course.

## 3. Discussion

Retained surgical item is an entity that has been in existence since the introduction of surgery into the armamentarium of medical treatment; it has a worldwide occurrence irrespective of the level of sophistication of medical practice, and it can complicate all arrays of surgical procedures. Retained surgical item is defined as surgical material (be it tools, supplies, or equipment) inadvertently left in patients by surgical provider and can cause harm [8]. Surgical items that may be retained range from sponges, needles/sharps, instruments, and those items classified as miscellaneous small objects like wire and tube [9]. Although cases with sponge/gauze are most reported in literatures [10], this case report found an instrument, a Kocher surgical clamp.

This patient remained asymptomatic for 15 years before incidental detection during routine evaluation for infertility. The authors have reported symptoms of RSI developing within hours to years after surgery, with a mean detection time of 21 days [6, 11, 12]. Symptoms are likely to be in the radar of fever, vomiting, sepsis/abscess, fistula, small bowel obstruction, visceral perforation, abdominal mass, granuloma formation, and gastrointestinal haemorrhage [13, 14]. As it was the case in this report, abdominal and pelvic cavities rank the highest location of RSI in 54% of cases followed by vaginal cavity and thoracic cavity in 22% and 7% of cases, respectively [6]. Known predisposing factors of RSI in this patient are open access surgery (laparotomy), emergent nature of the surgery, and obesity [6]. Other risk factors reported by authors are unplanned changes in surgical procedure, involvement of more than one surgical team, greater blood loss during the procedure, and multiple, complex, and lengthy surgeries [6, 7]. In this case report, the patient had a repeat laparotomy to retrieve the RSI (Kocher clamp) and postoperative recovery was good. Laparoscopic retrieval of RSI has been reported with excellent recovery [4]. The use of minimally invasive surgery in the management of this patient was not an option because of the availability and affordability of this type of surgical procedure in this part of the world.

Of utmost importance in reporting this case is to bring to the fore the conscious essence that must be inculcated towards preventing RSI in all operating rooms, notwithstanding the level of sophistication of medical practice. As a result of persistence in the occurrence of RSI, initiatives like NoThing Left Behind towards making RSI “a never happen event” and preventable best practices like Association Of Perioperative Registered Nurses (AORN's) perioperative guidelines recommended practices for sponge, sharps, and instrument counts are all efforts towards making RSI a rarity [9, 15]. Technological advancements are of equal importance in the prevention of RSI; some methods developed are computer assisted sponge count, radiofrequency detection system, and radiofrequency identification system [4]. Worthy of emphasis in the prevention of RSI are effective communication, coordinated adherence to best practice, and universal precautionary method in the operating room by stakeholders. In conclusion, a consistent and subconscious awareness by the surgical team that RSI is a possible complication in all surgical procedures should always be borne in mind, more especially when the surgery is associated with the labyrinth of a difficult, lengthy, and expansive surgical intervention.

## References

[1] Flores-Suarez R., Valle J. R.-D. (2010). A foreign body. *The New England Journal of Medicine*.

[2] National Quality Forum http://www.qualityforum.org/Home.aspx.

[3] National Patient Safety Agency Website http://www.npsa.nhs.uk/.

[4] Gibbs V. C. (2011). Retained surgical items and minimally invasive surgery. *World Journal of Surgery*.

[5] Kiernan F., Joyce M., Byrnes C. K., O'Grady H., Keane F. B. V., Neary P. (2008). Gossypiboma: a case report and review of the literature. *Irish Journal of Medical Science*.

[6] Gawande A. A., Studdert D. M., Orav E. J., Brennan T. A., Zinner M. J. (2003). Risk factors for retained instruments and sponges after surgery. *The New England Journal of Medicine*.

[7] Lincourt A. E., Harrell A., Cristiano J., Sechrist C., Kercher K., Heniford B. T. (2007). Retained foreign bodies after surgery. *Journal of Surgical Research*.

[8] Possover M. (2008). Gossypiboma in the pouch of Douglas. *The New England Journal of Medicine*.

[9] Association of Perioperative Registered Nurses (2010). Recommended practices for prevention of retained surgical items. *Perioperative Standards and Recommended Practices*.

[10] Sturdy J. H., Baird R. M., Gerein A. N. (1967). Surgical sponges: a cause of granuloma and adhesion formation. *Annals of Surgery*.

[11] Rappaport W., Haynes K. (1990). The retained surgical sponge following intra-abdominal surgery: a continuing problem. *Archives of Surgery*.

[12] Stoll A. (1988). Retained surgical sponge 40 years after laminectomy. Case report. *Surgical Neurology*.

[13] Kokubo T., Itai Y., Ohtomo K., Yoshikawa K., Iio M., Atomi Y. (1987). Retained surgical sponges: CT and US appearance. *Radiology*.

[14] Popoola A. A., Bello J. O., Ezeoke G. G., Adeshina K. T., Fadimu A. (2014). Concealed enterovesical fistula associated with forgotten intra-abdominal haemostat and intravesical towel. *Case Reports in Urology*.

[15] NoThing Left Behind http://www.nothingleftbehind.org/.

